# Sustained CD8+ T Cell Memory Inflation after Infection with a Single-Cycle Cytomegalovirus

**DOI:** 10.1371/journal.ppat.1002295

**Published:** 2011-10-06

**Authors:** Christopher M. Snyder, Kathy S. Cho, Elizabeth L. Bonnett, Jane E. Allan, Ann B. Hill

**Affiliations:** 1 Department of Molecular Microbiology and Immunology, Oregon Health and Sciences University, Portland, Oregon, United States of America; 2 School of Medicine and Pharmacology, The University of Western Australia, Crawley, Western Australia, Australia; University of Iowa, United States of America

## Abstract

Cytomegalovirus (CMV) is a β-herpesvirus that establishes a lifelong latent or persistent infection. A hallmark of chronic CMV infection is the lifelong persistence of large numbers of virus-specific CD8+ effector/effector memory T cells, a phenomenon called “memory inflation”. How the virus continuously stimulates these T cells without being eradicated remains an enigma. The prevailing view is that CMV establishes a low grade “smoldering” infection characterized by tiny bursts of productive infection which are rapidly extinguished, leaving no detectable virus but replenishing the latent pool and leaving the immune system in a highly charged state. However, since abortive reactivation with limited viral gene expression is known to occur commonly, we investigated the necessity for virus reproduction in maintaining the inflationary T cell pool. We inhibited viral replication or spread *in vivo* using two different mutants of murine CMV (MCMV). First, famcyclovir blocked the replication of MCMV encoding the HSV Thymidine Kinase gene, but had no impact on the CD8+ T cell memory inflation once the infection was established. Second, MCMV that lacks the essential glycoprotein L, and thus is completely unable to spread from cell to cell, also drove memory inflation if the virus was administered systemically. Our data suggest that CMV which cannot spread from the cells it initially infects can repeatedly generate viral antigens to drive memory inflation without suffering eradication of the latent genome pool.

## Author Summary

Cytomegalovirus (CMV) establishes life-long, asymptomatic infections in healthy people. Ongoing immune surveillance prevents viral disease but also results in the accumulation of large numbers of virus-specific T cells. The mechanisms by which the virus persists while stimulating such strong immune responses are unknown. We and others had hypothesized that periodic viral replication and spread to neighboring cells allowed CMV to replenish the pool of infected cells while stimulating virus-specific T cells to accumulate. In this manuscript, we have tested this model by blocking the replication or spread of murine cytomegalovirus (MCMV) and found, surprisingly, that accumulation of virus-specific T cells occurs independently of viral replication. Moreover, these T cells developed the terminal differentiated phenotype that is indicative of repeated antigenic stimulation. Thus, these data suggest that CMV can remain active and continuously stimulate the immune system, while avoiding immune-mediated clearance, without the capacity to spread from cell to cell.

## Introduction

Cytomegalovirus is a β-herpesvirus that establishes life-long, persistent infections in healthy people, and is associated with significant morbidity in immunosuppressed individuals. CMV infects a wide range of cells and tissues which, combined with the low levels of virus in a given tissue, has made the study of CMV latency extremely challenging. Long-term CMV carriage is characterized by viral latency in many organs, but the variety of cells that can harbor latent virus is unclear. Hematopoietic progenitor cells in humans [1] and liver sinusoidal endothelial cells in the mouse [2] have been shown to harbor latent virus, but in both species it is likely that other cellular sites also exist, and their relative importance is unknown (reviewed in [3], [4]). Latency is interrupted by repeated cycles of reactivation and occasional replication in discrete foci throughout the body; this is only occasionally detectable in mouse models. Abortive reactivation (viral gene expression that does not ultimately result in virion production) is common, at least in the mouse model and CD8+ T cells have been shown to contribute by preventing the cascade of lytic cycle gene expression from progressing past the immediate early (IE) or early (E) gene stages [5], [6]. Destruction of the infected cells by virus-specific CD8+ T cells seemed a likely mechanism to account for this block in viral gene expression, although this has not been shown. Thus, how the latent pool of virus is maintained through this process of reactivation and immune recognition remains unclear.

Throughout the life of the host, CMV infection is characterized by the presence of large numbers of virus-specific effector CD8+ T cells. In both murine and human CMV infections, these cells have been shown to increase in number after resolution of acute infection, and as a result the process has been called “memory inflation” [7]–[13]. In healthy human adults, an average of 5% of all CD8+ T cells are specific for CMV [14], and the frequencies can be even higher in experimentally infected mice. The frequencies of CMV-specific cells increase with advancing age and can ultimately result in distortions of the T cell compartment and large, dysfunctional clonal expansions [11], [15]–[20].

The dynamics of T cell-antigen interaction that lead to memory inflation are unknown. However, despite the absence of continuously detectable viral activity, the evidence suggests that memory inflation, and ultimately the distortion of the T cell compartment, is a direct result of persistent virus activity. First, the majority of CMV-specific CD8+ T cells display an end-stage differentiated effector phenotype, implying repeated antigenic stimulation (CD27^lo^, CD28^lo^, CD127^lo^, KLRG-1^+^) [12], [13], [21], [22]. Consistent with this interpretation, the frequency of cells with this phenotype in overt human CMV infection correlates with viral activity [23], [24]. Second, in the C57BL/6 mouse model, ongoing viral activity is particularly illustrated by CD8+ T cell recognition of an epitope encoded by the viral IE3 gene. These cells are barely detectable early in infection, begin to accumulate only several weeks after infection and ultimately come to dominate the chronic MCMV-specific T cell pool [10], [13]. Finally, our previous data showed that most inflationary cells in MCMV infected mice are unable to sustain their numbers through homeostatic or antigen-driven division and are destined to die, even in the presence of the persistent infection. However, the inflationary populations are continuously replenished by new virus-specific effector cells which differentiate from a pool of memory cells established early in infection [13], presumably in response to foci of reactivating virus.

The dynamic that allows for both repeated T cell stimulation and viral persistence is not understood. It is clear that T cells can recognize and limit viral reactivation before it proceeds to genome replication and full virion production [6]. If CD8+ T cells kill these infected cells, then the virus must be able to replenish the latently infected pool of cells in order to persist. Thus, the occurrence of infrequent, small foci of productive virus infection seemed to be the most likely explanation for the simultaneous occurrence of virus persistence and continuous immune stimulation. Virions produced in these foci could infect new cells and reestablish latency while the immune system recognized and cleared the originally infected cells. This model predicts both recurrent exposure to antigen, which would explain the high numbers of effector CD8+ T cells and persistence of the viral infection. We sought to test this model by drug blockade of viral DNA replication or by infecting with a variant of MCMV lacking the essential glycoprotein L (ΔgL) that is unable to spread from cell to cell. If persistent viral replication and infection of new cells were essential for the continued effector/effector memory CD8+ T cell response, we would expect CD8+ T cell memory inflation to be eliminated, the antigen-specific populations to contract, and the remaining cells to be largely quiescent memory cells. Indeed, this was what occurred in a mouse model of systemic Herpes Simplex Virus (HSV) infection, in which drug blockade of HSV DNA replication prevented CD8+ T cell memory inflation [25]. However, our data show that MCMV spread and/or replication is completely dispensable for memory inflation, here defined as the maintenance or accumulation of effector/effector memory CD8+ T cells specific for certain viral antigens. The spread defective ΔgL virus even elicited the delayed IE3-specific inflationary response, which first became detectable 8–12 weeks after systemic infection. Moreover, most responding T cells still gained expression of KLRG-1 and lost expression of the IL-7R (CD127), phenotypic hallmarks of inflationary T cells. These data indicate that a relatively small number of latently or persistently infected cells can repeatedly activate the immune system without loss of the viral genome.

## Results

### The thymidine kinase gene from HSV-1 renders MCMV extremely sensitive to acyclovir and famcyclovir

**Figure 1:**
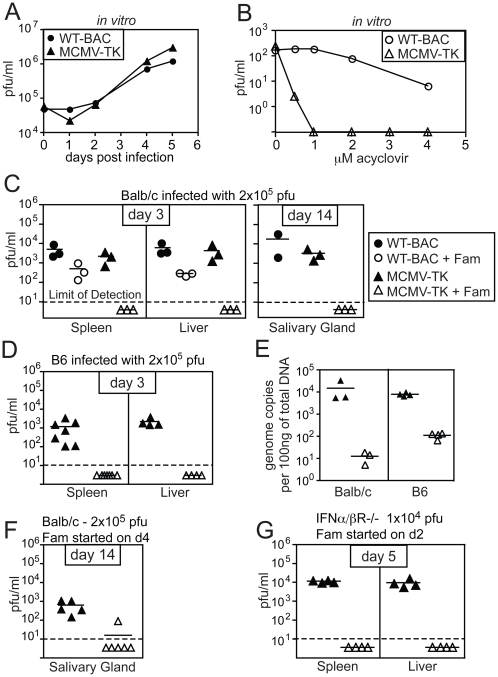
Replication of MCMV-TK is inhibited by acyclovir or famcyclovir. A) BALB-3T3s were infected with the indicated virus at a multiplicity of infection equal to 1 and infected cell lysate was taken on the indicated days for plaque assay. B) BALB-3T3s were infected with 200 pfu of MCMV-TK and treated with the indicated concentration of acyclovir. Plaques were counted 6 days later. C) Balb/c mice were left untreated or were treated with famcyclovir beginning 3 days before infection with wild-type MCMV or MCMV-TK. Viral titers were assessed by plaque assay in the spleens and livers 3 days after infection, or the salivary gland 14 days after infection. D) B6 mice were infected and treated as in C. E) Total splenic DNA from mice infected for 3 days and famcyclovir treated or untreated, was tested for the presence of MCMV by quantitative PCR. F) Famcyclovir treatment was initiated on d4 post infection of BALB/c mice with MCMV-TK. Viral titers in the salivary gland were measured by plaque assay on day 14. G) IFNα/βR−/− mice were treated with famcyclovir from day 2 to day 5 post infection. Viral titers were measured by plaque assay as above.

To investigate the amount of virus activity needed to sustain memory inflation, we needed to be able to inhibit viral replication more completely than is possible with available antiviral drugs, to which MCMV is only partially sensitive. Acyclovir is a guanosine analogue that acts as a DNA chain terminator when it is phosphorylated by herpes simplex virus thymidine kinase (TK) [26]. However, MCMV lacks TK [27], [28] and is thus relatively insensitive to acyclovir and its derivatives. To render MCMV sensitive to acyclovir, we replaced the early gene *m157* of MCMV with the *TK* gene from HSV-1 to render the recombinant virus sensitive to acyclovir and its derivatives. Deleting *m157* also rendered the virus resistant to NK cell control in C57BL/6 mice [29], [30], which resulted in increased viral titers during acute infection but did not substantially affect the chronic CD8+ T cell response (Cho et. al., manuscript in preparation). MCMV-TK grew with normal kinetics *in vitro* (Figure 1A), but was exquisitely sensitive to acyclovir compared to wild-type MCMV (Figure 1B). To test whether we could block replication of the TK-expressing virus *in vivo*, we used famcyclovir, an orally available analogue of acyclovir, that effectively blocks HSV-1 replication in mice [25], [31], [32]. BALB/c mice (in which deletion of *m157* does not affect MCMV titers) or C57BL/6 mice were left untreated or were treated continuously with famcyclovir in their drinking water beginning 3 days before infection with wild-type or MCMV-TK viruses. While famcyclovir reduced the replication of wild-type virus in the spleens and livers of treated animals, it completely prevented the detection of infectious MCMV-TK (Figure 1C and D). Moreover, treatment reduced the viral genome copy number to undetectable levels (at least 100 to 1000 fold) in B6 and BALB/c mice (Figure 1E), indicating that famcyclovir effectively blocked viral DNA replication. To determine whether famcyclovir could also inhibit an ongoing infection, treatment was delayed until day 4 of the infection in BALB/c mice, which corresponds to the peak viral burden in the spleen and liver (not shown). No replicating virus could be detected in the salivary glands of 5 out of 6 treated mice 10 days later (d14 post infection - Figure 1F). Thus, famcyclovir could control an ongoing infection and prevent viral spread to the salivary gland, which is a major site of viral replication after the first week of infection. Additionally, famcyclovir treatment from days 2 to 5 was sufficient to block ongoing viral replication in highly susceptible Type I IFN receptor knock out mice (Figure 1G). Together these data show that MCMV-TK is extremely sensitive to the antiviral drug famcyclovir and that oral treatment is sufficient to block systemic viral replication and spread.

### Famcyclovir treatment of MCMV-TK infected mice does not diminish the frequency of MCMV-specific CD8+ T cells

**Figure 2:**
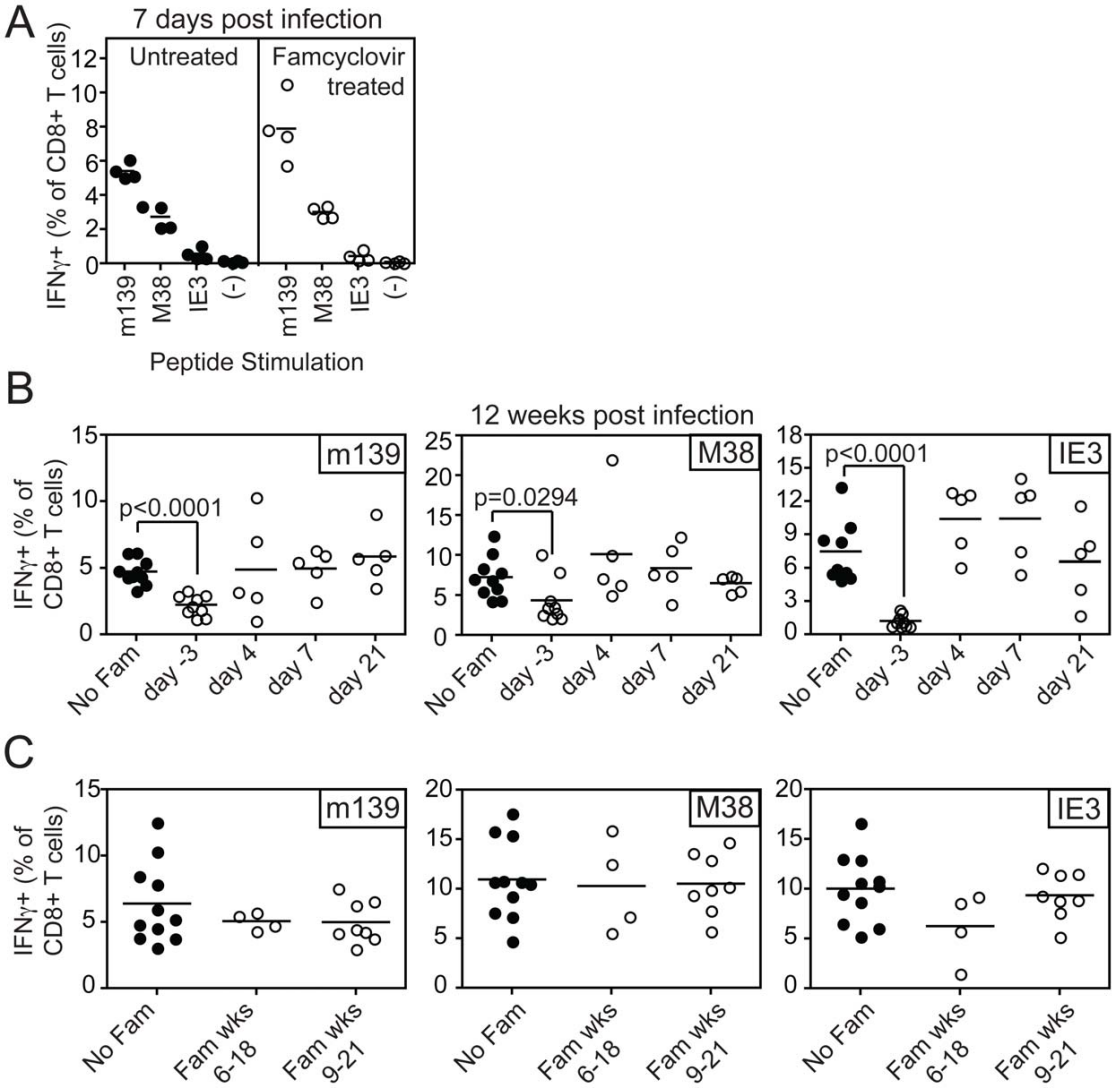
Inhibiting viral replication with famcyclovir does not reduce the size of virus-specific T cell populations. A) B6 mice were left untreated or treated with famcyclovir from 3 days before infection with MCMV-TK. CD8+ T cell responses were measured in the peripheral blood 7 days post infection by intracellular cytokine staining after stimulation with the indicated peptides. B) Famcyclovir treatment was initiated at the indicated day and mice were infected on day 0 with MCMV-TK. Virus-specific T cells were measured 12 weeks after infection in the peripheral blood by intracellular cytokine staining after stimulation with the indicated peptides. C) Famcyclovir treatment of MCMV-TK infected mice was initiated in the 6th or 9th week after infection and maintained for 12 weeks thereafter. Virus-specific CD8+ T cells were measured as in A and B, at the end of the time course.

To test the impact of blocking viral replication on the accumulation or maintenance of MCMV-specific inflationary CD8+ T cells, famcyclovir treatment was initiated at various times before or after MCMV-TK infection. CD8+ T cell responses to the three dominant inflationary epitopes, encoded by m139, M38 and IE3, were monitored in the peripheral blood. Pre-treatment of mice with famcyclovir for 3 days before infection did not inhibit the primary immune response to inflationary antigens (Figure 2A). However, if famcyclovir treatment was begun 3 days before infection and maintained continuously for 12 weeks after infection, we observed a significantly reduced frequency of inflationary CD8+ T cells at the end of the time course (designated as day −3, Figure 2B). In particular, the late-arising response to IE3 was barely detectable. Thus, CD8+ T cell memory inflation was blocked by pre-treatment with famcyclovir. Strikingly however, if famcyclovir treatment was started 4, 7 or 21 days after infection and maintained until 12 weeks post infection, there was no significant reduction in the frequency of inflationary CD8+ T cells that had accumulated during this time (Figure 2B). Similarly, if famcyclovir treatment was started in the 6th or 9th week after infection and maintained for the subsequent 12 weeks into the chronic phase of infection, there was no significant difference in the frequency of inflationary CD8+ T cells (Figure 2C). These data seemed at odds with the presumption that memory inflation depends on viral replication: they suggested that, once infection has been established, further virus replication was not needed to maintain memory inflation. However, we can not exclude the possibility that famcyclovir inhibition of MCMV replication was incomplete, perhaps especially in some critical cells or organs.

### Spread-defective MCMV is also capable of driving CD8+ T cell memory inflation

To more stringently examine whether viral replication is needed to maintain memory inflation, we used a recently characterized recombinant MCMV lacking the essential glycoprotein L (ΔgL) [33]. The viral gL is essential for incorporation of the gH/gL complex into the viral envelope, which is needed for entry into cells (reviewed in [34]). This virus is propagated on a complementing cell line that provides gL in *trans*, generating virions that can infect cells and produce all classes of viral proteins and replicate viral DNA. However, progeny virions lack gH/gL and are unable to infect new cells *in vitro* or *in vivo* [33], making this gL-deleted virus spread-defective.

**Figure 3:**
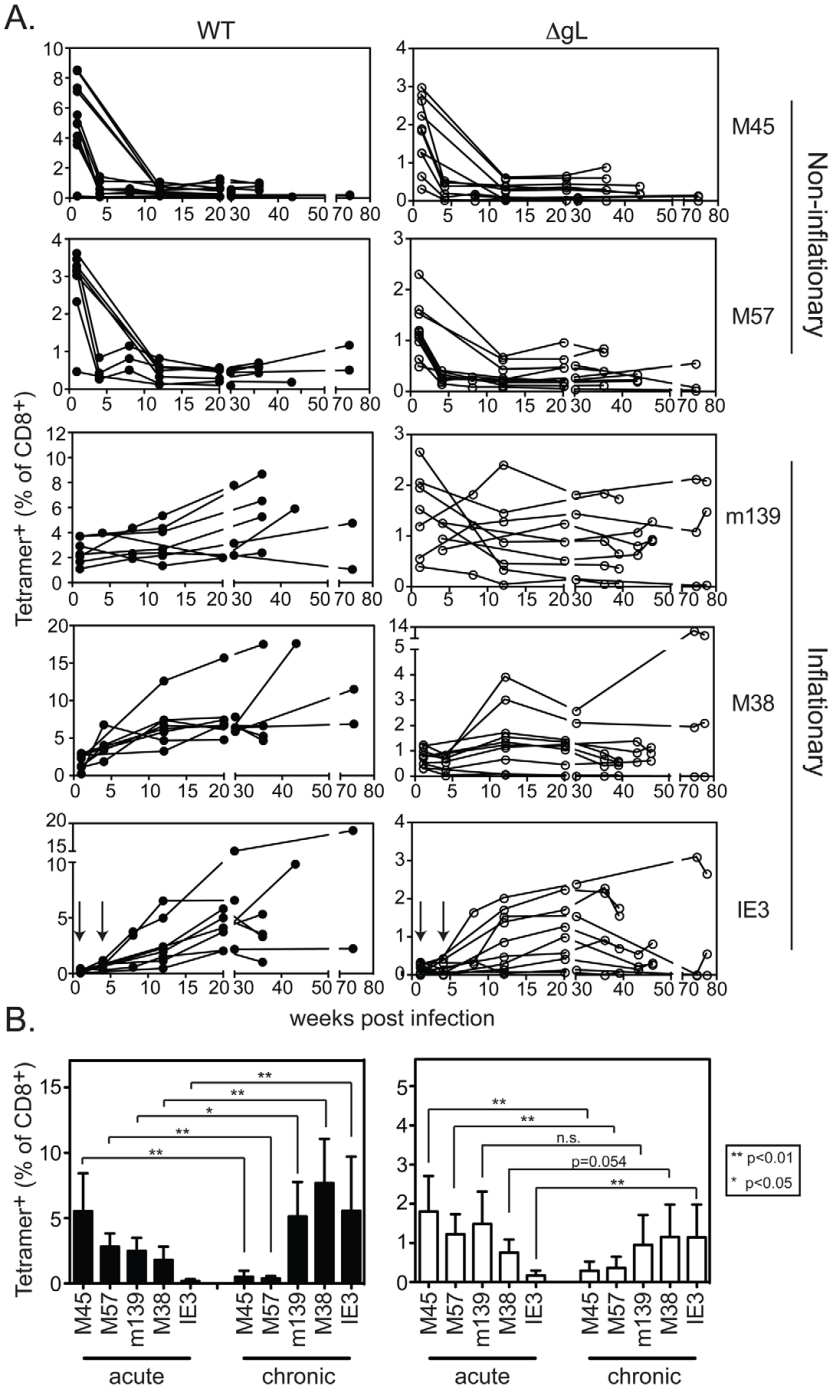
Spread-defective MCMV drives CD8+ T cell memory inflation. A) Mice were infected with WT-BAC or wild-type K181 MCMV (filled symbols, n = 8, labeled “WT” for wild-type) or ΔgL MCMV (n = 10, open symbols) and virus-specific CD8+ T cells were measured in the peripheral blood by tetramer staining at the indicated times post infection. Each line represents an individual mouse followed over time. Data was combined from 3 independent experiments. B) The mean and standard deviation of the responses in all infected mice was compared at acute (1 week post infection) or chronic time points. Data for the chronic time point is an average of antigen-specific frequencies 20, 30 or 36 weeks post infection (each mouse was only counted once depending on which time point data was collected). Statistical significance was determined by the student's t-test for paired data.

C57BL/6 mice were infected i.p. with wild-type or ΔgL MCMV, and the CD8+ T cell response was followed in the peripheral blood over time. As previously described, acute ΔgL MCMV infection induced the broad range of CD8+ T cell responses elicited by acute wild-type infection ([33] and Figure 3). The responses to ΔgL infection were substantially smaller than after wild-type infection (note the different y-axis scales), but nevertheless followed a strikingly similar pattern. In both wild-type and ΔgL infections, M45 and M57-specific responses contracted after day 7 while m139-, M38- and IE3-specific responses increased or were maintained (Figure 3). The responses were not identical. While m139- and M38-specific T cells accumulated in most mice infected with wild-type MCMV, similar accumulation only occurred in some mice after ΔgL infection (Figure 3A). However, on average, these responses were maintained after ΔgL infection and thus m139- and M38-specific T cells became co-dominant upon contraction of M45 and M57-specific T cells (Figure 3B). Notably, T cells specific for IE3, which are barely detectable in the first 4 weeks after infection with either virus (arrows in IE3 plot), eventually accumulated in 8 of 10 ΔgL infected mice. The two animals that did not develop an IE3-specific T cell response after ΔgL infection also failed to maintain the m139- or M38-specific responses. We note that the overall size of the inflationary populations is lower after ΔgL infection than in wild-type infected mice, which likely reflects the reduced latent viral burden these animals. However, in both infections, the maintenance or accumulation of m139-, M38- and IE3-specific T cells resulted in a similar change in the immunodominance hierarchy from acute to chronic time points (Figure 3B).

**Figure 4:**
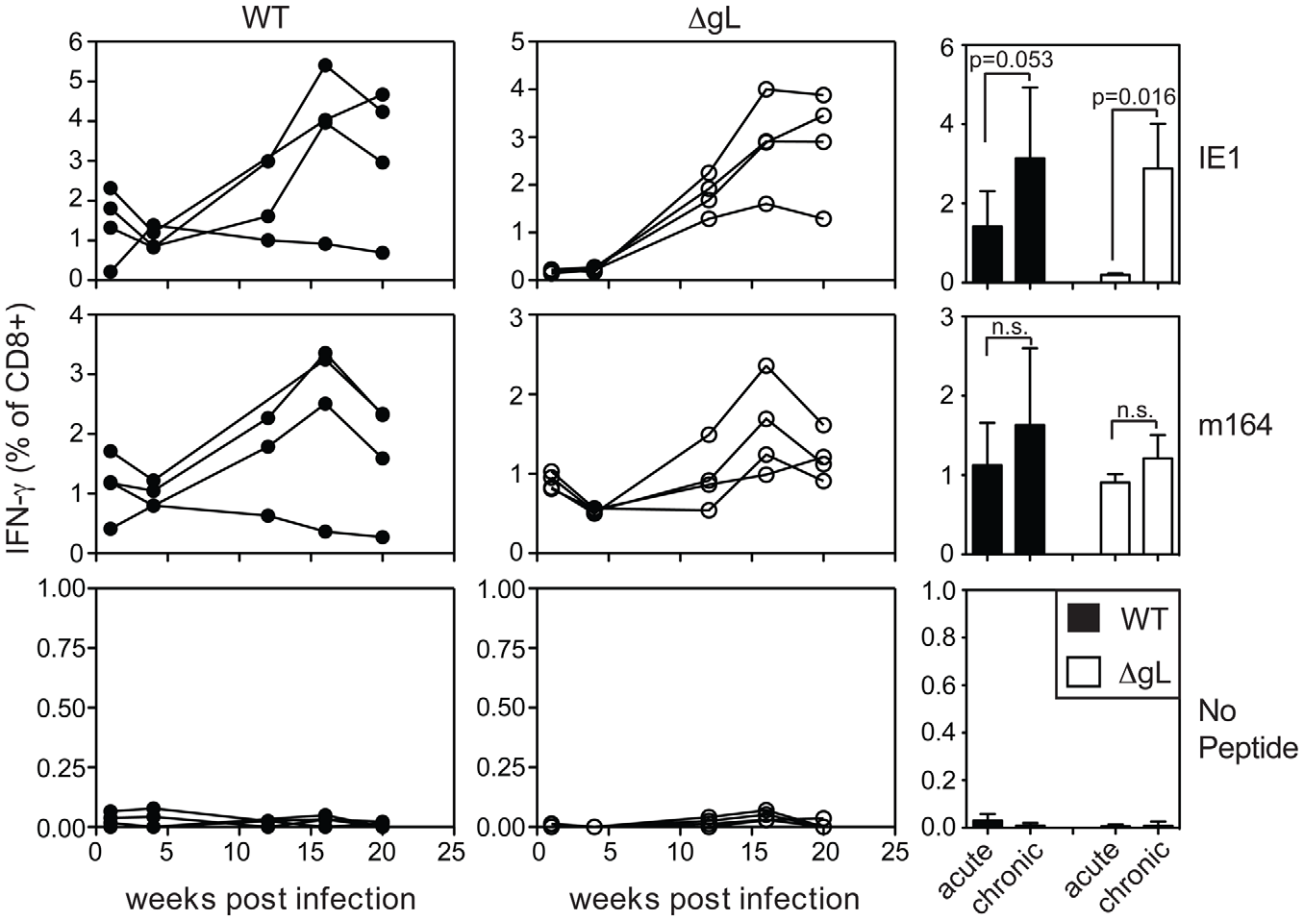
Spread-defective MCMV drives CD8+ T cell memory inflation in BALB/c mice. Mice were infected with wild-type K181 MCMV (filled symbols, n = 4, labeled “WT” for wild-type) or ΔgL MCMV (n = 4, open symbols) and virus-specific CD8+ T cells were measured in the peripheral blood at the indicated time post infection by the ability to produce IFN-γ after stimulation with the indicated peptides. Each line represents an individual mouse followed over time. The bar graphs (right-most column) represent the mean and standard deviation for data collected at acute (1 week) or chronic (20 weeks) time points. Statistical significance measured by the student's t-test for paired data.

We also tested ΔgL MCMV in the common BALB/c model of infection, in which T cells specific for two epitopes from the IE1 and m164 proteins are co-dominant in acute infection and also undergo memory inflation. Here, the inflationary response to ΔgL infection was even more impressive, reaching levels that were similar to inflation after wildtype infection (Figure 4). Interestingly, in contrast to wildtype infection, IE1-specific T cells were virtually undetectable 1 and 4 weeks after ΔgL infection. However, they accumulated at later times and eventually dominated the response, in a pattern highly reminiscent of the IE3 response in C57BL/6 mice. Together, the maintenance or accumulation (memory inflation) of virus-specific CD8+ T cells after ΔgL infection of B6 and BALB/c mice suggest ongoing viral antigen production for at least 20 weeks (BALB/c mice) or 36–74 weeks (B6 mice). Whether this activity will be life-long remains to be determined.

**Figure 5:**
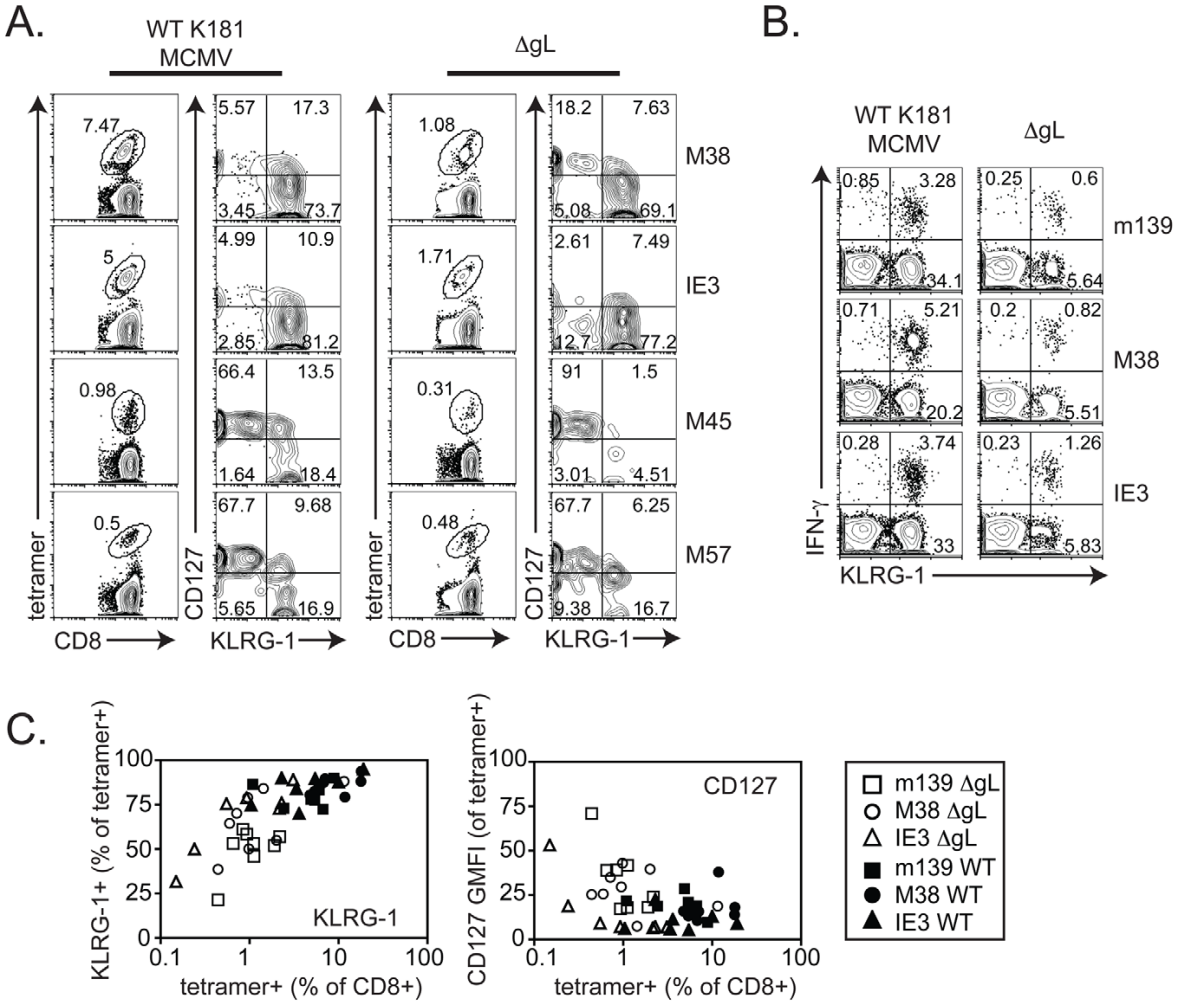
CD8+ T cell differentiation correlates with inflation. A) 20 weeks after infection, virus-specific CD8+ T cells in the peripheral blood were identified by tetramer staining and cells were co-stained with antibodies specific for CD127 (IL-7Rα) and KLRG-1. B) 25 weeks after infection with wild-type or ΔgL MCMV, virus-specific T cells from the peripheral blood were measured by intracellular cytokine staining and co-stained with antibodies specific for KLRG-1. C) The phenotype of peripheral blood, inflationary T cells (m139-, M38- or IE3-specific) from the three experiments shown in Figure 3 is combined. Data correspond to blood drawn at 36, 43 or 74 weeks after infection. Populations that comprised less than 0.1% of all CD8+ T cells were assumed to result from non-specific tetramer staining (based on uninfected control mice) and were not graphed. Shown is the percentage of cells expressing KLRG-1 (left panel) and the geometric mean fluorescence of CD127 (right panel) within the tetramer+, CD8+ T cell population.

Cells with inflationary specificities, whether measured by tetramer staining (Figure 5A) or by their ability to produce IFN-γ upon stimulation (Figure 5B) were phenotypically similar in all B6 mice regardless of the virus used for infection, with the majority of m139, M38- and IE3-specific cells expressing high levels of the inhibitory molecule KLRG-1 and low levels of the IL-7Rα chain (CD127) and co-stimulatory molecule CD27 (Figure 5 and data not shown). This phenotype is thought to result from repeated antigen exposure [21], [22], [35], [36] and clearly contrasts with the phenotype of M45- and M57-specific T cells, which did not inflate in any animal (Figure 5A). Importantly, the phenotype of the inflationary populations correlated with the extent of T cell accumulation such that populations that were reduced in frequency also tended to express more CD127 and contain fewer cells expressing KLRG-1 (Figure 5C). Similar data have been obtained in humans infected with HCMV [23], [24], suggesting a direct relationship between viral activity, the size of inflationary populations and the degree of T cell differentiation.

**Figure 6:**
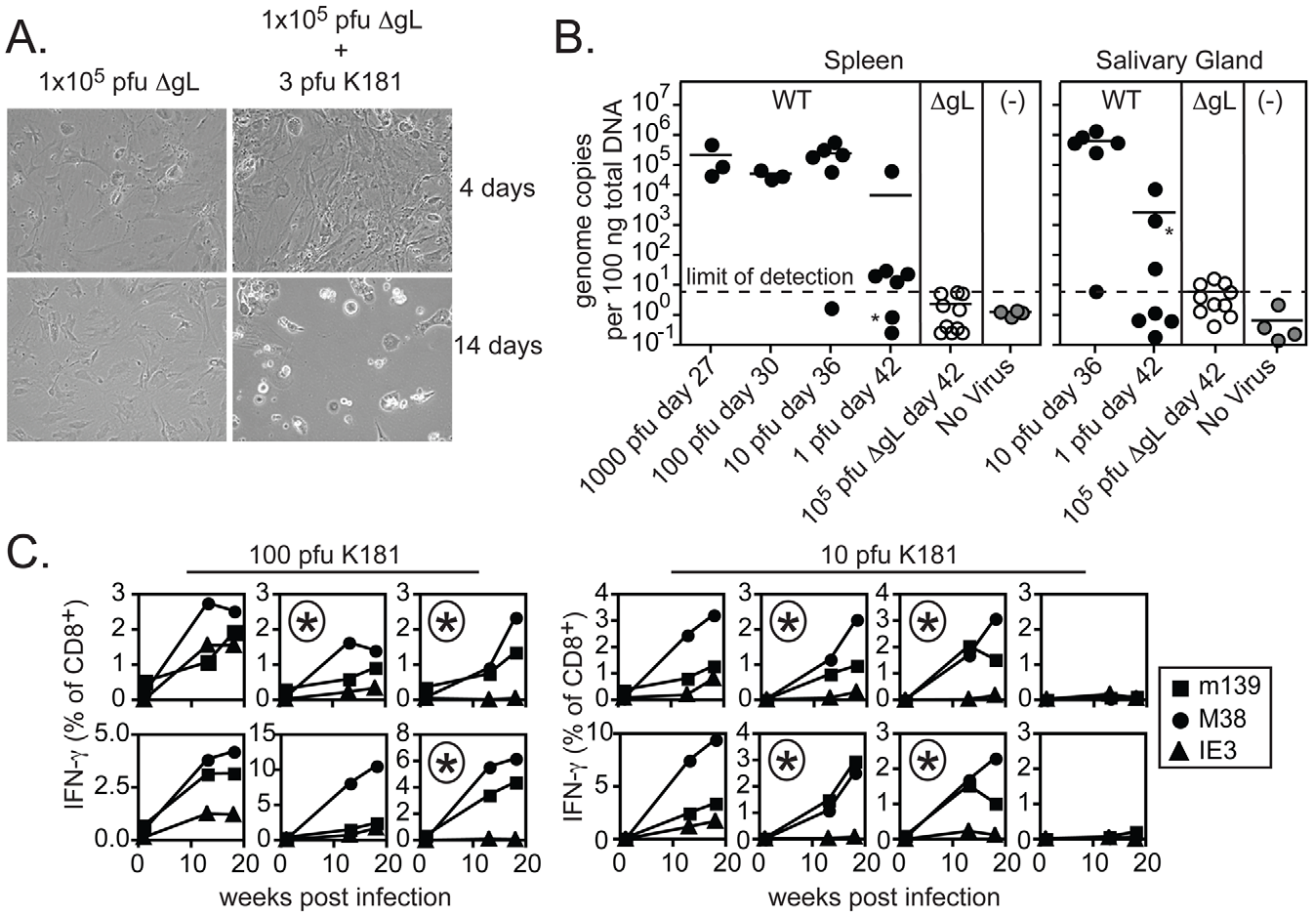
Contaminating spread-competent MCMV cannot account for CD8+ T cell memory inflation. A) MEFs were infected with 1×10^5^ pfu of ΔgL with or without 3 pfu of wild-type K181 MCMV. Shown are representative images of infected cultures taken 4 and 14 days after infection. Data is representative of at least 8 independent experiments. B) BALB/c-SCID mice were infected with the indicated amounts of wild-type K181 MCMV or 1×10^5^ pfu of ΔgL MCMV and sacrificed at the indicated day post infection. Uninfected control samples were derived from naïve C57BL/6 mice. Viral DNA was quantified in the spleens and salivary glands by qPCR. Data was combined from 2 independent experiments. One mouse infected with a single pfu of wild-type virus displayed detectable viral DNA in the salivary gland, but not the spleen (indicated by the asterisks). A correlation was found between the salivary gland and spleen in all other mice. The limit of detection in these assays was estimated to be 30 copies of viral DNA per 500 ng of total DNA as measured by a standard curve of the viral E1 gene cloned into a plasmid. C) B6 mice were infected with 10 or 100 pfu of wild-type K181 MCMV and antigen-specific T cells were tracked in the peripheral blood by tetramer staining. Each box shows the T cells specific for m139, M38 and IE3 within a single mouse. The boxes marked with an asterisk indicate mice in which IE3-specific T cells failed to inflate despite accumulation of m139- and M38-specific T cells. Data was combined from 2 independent experiments.

These results suggest that ΔgL MCMV persists and remains antigenically active, driving late-developing IE3- and IE1-specific T cell responses without the ability to spread from the first cells infected. Because of the profound implications of these results, it was especially important to verify that the viral stock injected did not contain any virus that had recovered gL-expression and thus, the ability to spread from cell to cell. For example, it is formally possible for the gL gene to be restored through homologous recombination with the complementing cell line in a fraction of virions. Such a rescued contaminant would have had to occur consistently, since the data shown in Figures 3, 4 and 5 represents four independent experiments using three independently produced ΔgL preparations. Nevertheless, to formally test whether the ΔgL might be rescued at a frequency that could account for our results, we performed a series of *in vitro* and *in vivo* infections with each of the virus stocks used for the experiments shown in Figures 3, 4 and 5. First, cultures of non-complementing murine embryonic fibroblasts (MEFs) were infected with at least 1×10^5^ and up to 1×10^6^ plaque forming units (pfu) of ΔgL virus. Following infection, cultures were maintained for 4 weeks without evidence of viral growth (Figure 6A and data not shown). In contrast, just 3 pfu of wild-type MCMV added to the ΔgL infected cultures was sufficient to kill all of the cells within 2 weeks (Figure 6A). Second, to test whether any fraction of the ΔgL virus was able to spread *in vivo*, BALB/c-SCID mice were infected with 1×10^5^ pfu of the ΔgL virus or varying amounts of wild-type MCMV. While just 10 pfu of wild-type virus overwhelmed these mice in 5 weeks, no virus was detectable above background in the spleens or salivary glands of ΔgL infected mice after 6 weeks (n = 10 mice, Figure 6B and data not shown). Together, these data show that any spread-competent virus contaminating our preparations could maximally comprise between 0.001% (1 per 1×10^5^) and 0.0001% (1 per 1×10^6^) of all viruses in our preparations. Since we used 1×10^5^ pfu of the ΔgL virus to infect mice for the experiments in Figures 3, 4 and 5, at most we would expect 1 pfu of contaminating, spread-competent virus in each infection. Finally, to determine whether our results are consistent with a small, contaminating population of spread-competent MCMV, additional mice were infected with 100 or 10 pfu of wild-type K181 MCMV (K181 is the wild-type, parental strain used to generate ΔgL). Most mice infected with these low doses of wild-type virus showed evidence of memory inflation within the m139- and M38-specific CD8+ T cell populations (12 out of 14 mice, shown individually - Figure 6C). Strikingly however, of the 12 mice that developed m139- and M38-specific responses, 7 failed to develop an inflating IE3-specific CD8+ T cell response (individual mice marked with an asterisk, Figure 6C). This contrasts with the results obtained by infection with 1×10^5^ pfu of the ΔgL virus, which induced inflation of IE3-specific CD8+ T cells in all mice that responded to m139 and M38 antigens and 8 out of 10 total mice (Figure 3). Together, these data suggest that the memory inflation induced by ΔgL infection cannot be explained by a small number of contaminating, spread-competent viruses.

### Spread-defective MCMV must infect host cells in order to drive CD8+ T cell memory inflation, but cannot be restricted to the footpad

**Figure 7:**
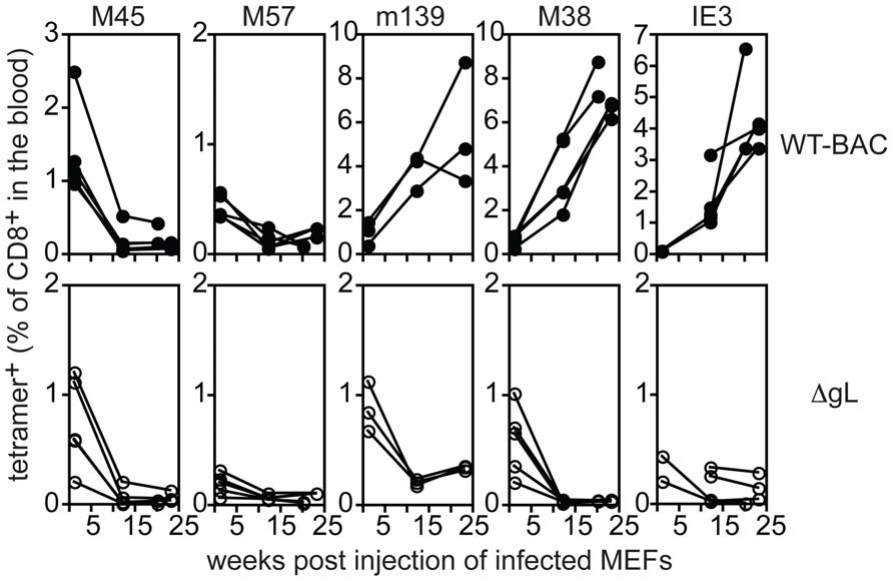
ΔgL MCMV must infect a cell population *in vivo*. B6 MEFs were infected with wild-type or ΔgL MCMV *in vitro*, washed in a citrate buffer to strip infectious virions that had not entered the cells, and then injected into naïve B6 mice. Antigen-specific T cell responses were measured in the peripheral blood by tetramer staining. Data was combined from 2 independent experiments and each line represents an individual mouse.

Since the ΔgL virus can only undergo one round of infection, we reasoned that viral persistence and CD8+ T cell memory inflation would be prevented if we “spent” the single infectious cycle on fibroblasts *in vitro*. To this end, murine embryonic fibroblasts (MEFs), which do not express the glycoprotein L, were infected with wild-type or ΔgL viruses *in vitro*. Following infection, the cells were washed stringently in a citrate buffer to remove any infectious virions that remained in the culture [33]. The absence of infectious virions associated with the infected fibroblasts was confirmed by plaque assay on the complementing gL-expressing cell line (not shown). The cells were then injected i.p. into mice, and CD8+ T cell responses in peripheral blood were measured by tetramer staining (Figure 7). Importantly, although primary CD8+ T cell responses were elicited in all animals, memory inflation only occurred in mice that received wild-type virus-infected MEFs. The primary response to ΔgL-infected fibroblasts was presumably driven by cross-presentation of antigen from dying cells, as we have previously suggested [33]. However, without the ability to directly infect cells *in vivo*, ΔgL did not drive memory inflation. This supports the idea that the ΔgL virus cannot spread from the first cells infected and suggests that memory inflation is critically dependent on the ability of the virus to access cells in the animal that are capable of maintaining latent/persistent infection.

**Figure 8:**
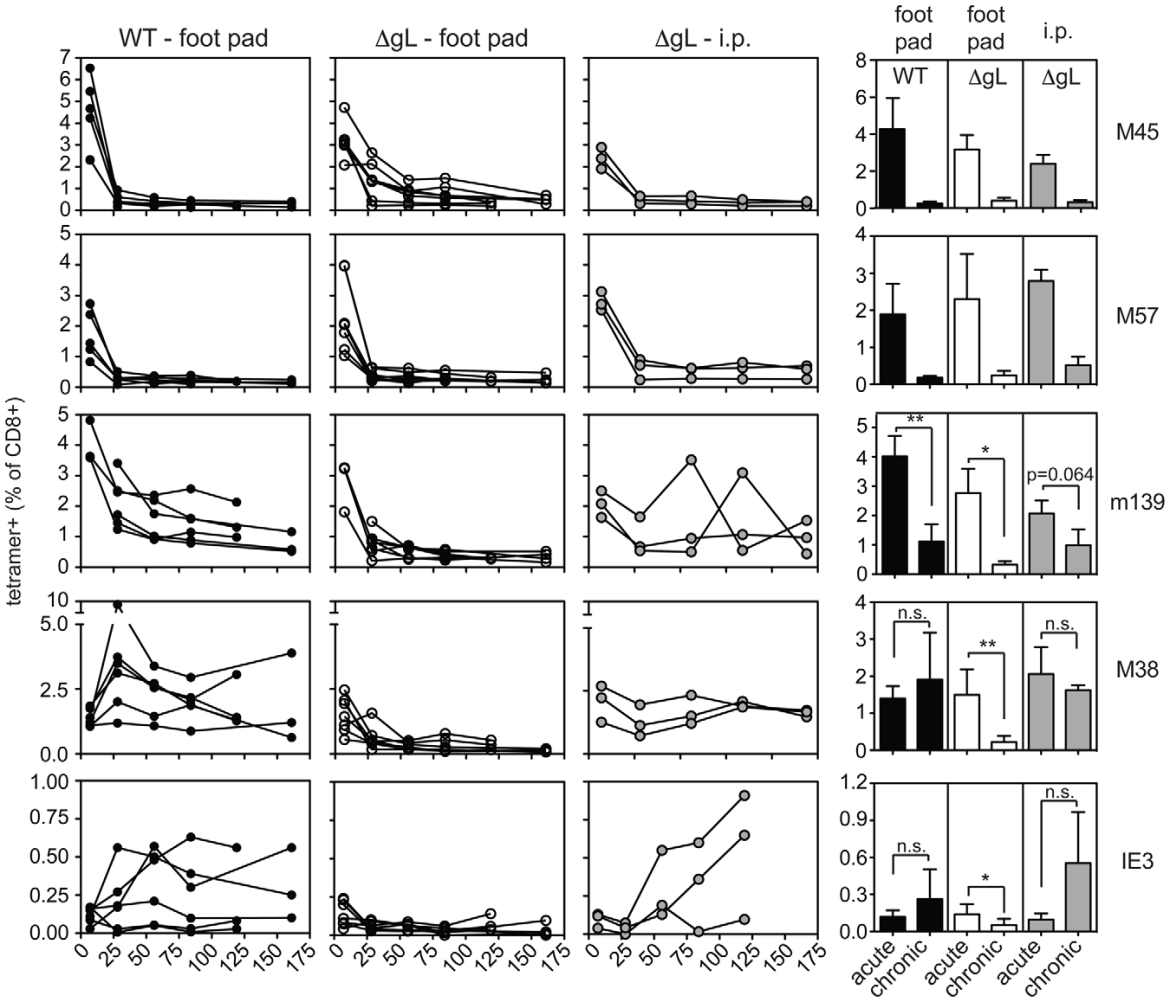
Foot pad injection of ΔgL MCMV fails to result in maintenance or inflation of m139-, M38- or IE3-specific CD8+ T cells. Antigen-specific T cell responses were measured in the peripheral blood by tetramer staining after foot pad infection of wild-type MCMV or ΔgL MCMV or intraperitoneal infection of the same amount of ΔgL MCMV. Data was combined from 2 independent experiments and each line represents an individual mouse. The right-most column shows the mean and standard deviation of the data collected from all mice at acute (1 week post infection) or chronic time points. Data for the average chronic response was drawn from the final time point measured for each mouse - 119 or 161 days post infection. Statistical significance was determined by the student's t-test for paired data.

In each of the above experiments, the ΔgL virus was administered by intraperitoneal injection, from which the virus gains access to the blood *via* the mediastinal lymphatics and within the first few hours infects a variety of cell types in the mediastinal lymph nodes, liver and spleen [37]. We wondered whether alternate routes of infection, with limited cell types available for immediate infection, might change the ability of the virus to persist and drive CD8+ T cell memory inflation. The hind foot pad is a commonly used site for experimental MCMV infection. In our hands, foot-pad injection of C57BL/6 mice with wild-type virus resulted in less CD8+ T cell accumulation than intraperitoneal infection (Figure 8, left column). Nonetheless, m139- and M38-specific T cell responses clearly remained elevated in comparison to non-inflationary M45- and M57-specific T cell responses. IE3-specific T cells were poorly elicited by foot-pad injection, but were detected in about half of the mice infected with wild-type virus. In contrast, foot-pad injection of the ΔgL virus yielded no detectable maintenance of any MCMV-specific populations, despite the fact that M45-, M57-, m139- and M38-specific T cells were all primed and readily detectable 1 week after infection (Figure 8, 2nd column from left). This is best illustrated by the M38-specific response, which is clearly maintained after foot pad injection of the wild-type virus, but not the ΔgL virus. In addition, IE3-specific T cells were never detected in mice infected with the ΔgL virus via the foot pad. Control mice infected with an identical dose of ΔgL virus delivered via the intraperitoneal route displayed maintenance or inflation of m139-, M38- and IE3-specific T cells in a pattern that mimicked the wild-type infection over the 12 week time frame (Figure 8, 3rd column from the left). Together, these results suggest that systemic inoculation of a spread-defective MCMV, but not a localized foot pad infection, allows for CD8+ T cell memory inflation. We conclude that maintaining or accumulating virus-specific CD8+ T cells after priming is not dependent on periodic viral replication during chronic infection. This implies that latently or persistently infected cells can repeatedly stimulate the immune system while avoiding clearance.

## Discussion

CMV latency and reactivation remains poorly understood, in part because the virus infects so many cell types and persists at vanishingly low levels (reviewed in [3]). Molecular studies have clearly shown that limited viral gene expression occurs in the absence of viral replication during chronic infection and that CD8+ T cells can recognize the products of these genes [5], [6], [38], [39]. The accumulation of effector/effector memory CD8+ T cells specific for CMV, which we and others call memory inflation, is yet another indicator of ongoing antigenic expression and viral persistence. However, it has never been clear whether CD8+ T cell recognition of reactivating virus resulted in clearance of the infected cells. If so, one might predict that the virus must produce infectious virions periodically, even at extremely low levels or in a privileged site, in order to maintain the latent genome pool in the face of a potent CD8+ T cell response. Here we show that ΔgL-MCMV, which cannot spread beyond the first cells infected, nonetheless drives the accumulation of virus-specific CD8+ T cells. This includes the late accumulation of IE3-specific T cells in B6 mice as well as delayed, but substantial accumulation of IE1-specific T cells in BALB/c mice. Importantly, these virus-specific T cells expressed the terminal-effector phenotype that is indicative of repeated antigenic stimulation. Thus, our data strongly suggest that MCMV can both persist and remain antigenically active after only a single round of productive infection.

The fact that replication-blocked and spread-defective MCMV were immunogenic upon initial infection was not surprising, since many different replication-deficient viruses are known to prime robust CD8+ T cell responses. Moreover, the idea that spread-defective MCMV might establish latency is not new, since this has been described previously for α- and γ-herpesviruses [40]–[43] as well as for temperature sensitive mutants of MCMV [44]. However, nothing in the literature predicted the fact that this latent virus would continue to robustly stimulate CD8+ T cell responses without being rapidly eliminated, even though it lacks the ability to replenish the pool of latently infected cells.

How can ΔgL be highly immunogenic, clearly visible to the immune system, but not eradicated by the immune system? The answer to this puzzle must lie in the nature of CMV latency or persistence and the cells that produce viral antigens, both of which remain poorly understood. It is likely that CMV can establish latency in a variety of cell types. In mice, stromal cells, including sinusoidal endothelial cells in the liver, have been implicated as the main sites from which latent virus can be reactivated [2], [45]. Latent human CMV can be found in CD34+ hematopoietic stem/progenitor cells, and it reactivates as these cells differentiate into dendritic cells [46], [47]. Similarly, it was shown many years ago that MCMV establishes a latent or persistent infection in undifferentiated cells in vitro and reactivates upon differentiation [48]. The phenomenon of latency in undifferentiated cells and replication in differentiated cells may be a common theme in CMV latency. In our study, a relatively small amount (10^5^ pfu) of ΔgL was injected, and viral DNA was undetectable even in SCID mice (Figure 6). However, our study suggests that at least one site of latency is capable of maintaining the viral genome without being replenished by viral replication and infection of new cells, and is simultaneously able to robustly stimulate CD8+ T cells. It is possible that latency in stem/progenitor cells could explain this. Since progenitor cells can divide asymmetrically during differentiation, this could allow the virus to persist in an antigenically silent state in the undifferentiated daughter cell, but stimulate the immune system during reactivation in the differentiated daughter cell. An alternative possibility is that latently or persistently infected cells may be recognized by the immune system, but resistant to immune-mediated lysis, as is the case for HSV-infected neurons [49]. This latter possibility would indicate a cell with unusual immunological properties. Neurons are highly immune-privileged. Long thought to be invisible to the immune system, it is now clear that neurons can be seen, but are protected from damaging immune mechanisms. However, they are not immune stimulatory: they do not prime an immune response, and the CD8+ T cells that surround HSV-infected neurons do not undergo memory inflation. The cells that harbor latent MCMV would not only need to be resistant to lysis, but they would have to also possess the ability to drive differentiation and proliferation of inflationary CD8+ T cells. Dendritic cells are not a likely candidate, as they are the only cells that we found to be readily lysed by CD8+ T cells specific for inflationary epitopes, despite viral inhibition of antigen presentation (figure 2 in [50]).

It is obviously important to identify the cells in which ΔgL-MCMV established latency. To date, we have found no evidence of viral RNA in hematopoietic stem cells. Additional possibilities include subcapsular macrophages in the mediastinal lymph nodes, reticular fibroblasts in the spleen, and hepatocytes in the liver, all of which are infected by MCMV within 8 hours after i.p. inoculation [37], but the latently infected cell could be a minor, as yet unnoted subset. Further investigation will be needed to determine whether any of these cells harbor latent ΔgL DNA and whether they can stimulate CD8+ T cells. However, it is unlikely that all of these cells support CMV latency, and since the memory inflation reported here resulted from an infection with only 10^5^ pfu of ΔgL virus, we are searching for a tiny needle in a large haystack.

Our studies with MCMV-TK infected mice treated with famcyclovir yielded analogous results to those infected with the ΔgL virus with one notable exception: whereas a single cycle of ΔgL infection elicited memory inflation, pretreatment of mice with famcyclovir, which should restrict the virus to the first round of infection, substantially blunted memory inflation. This finding is not entirely unexpected if we assume that any mechanism of viral persistence would require that CMV be capable of multiplying its genome. While the ΔgL virus can replicate its genome after the initial infection, famcyclovir blocks viral DNA replication of the MCMV-TK virus. Thus, pretreatment with famcyclovir would prevent the initial amplification of the viral genome even within the first cells infected. However, allowing the MCMV-TK virus to establish infection for just 4 days before initiating famcyclovir treatment resulted in normal CD8+ T cell accumulation. It is interesting that our famcyclovir experiments returned different results to those recently reported in HSV infection of mice, where famcyclovir, even when administered several days after infection, prevented memory inflation of CD8+ T cells responding to the HSV-gB epitope [25]. This difference may be due to the fact that expression of HSV-gB, a late protein, is inhibited by famcyclovir [31], while expression of the immediate-early and early genes encoding MCMV inflationary epitopes should be unaffected by the drug. Alternatively, different cellular sites and mechanisms of latency for the two viruses may explain the discrepancy.

These results also have practical implications for the use of CMV as a vaccine vector. Louis Picker and colleagues recently showed that a CMV-based vaccine elicited potent protection in a monkey model of AIDS, a result the authors attribute to the large effector memory (inflationary) CD8+ T cell response [51], [52]. We and others are also pursuing CMV as a vaccine vector for cancer. However, use of a live, replicating herpesvirus-based vaccine raises safety issues. We have recently shown that cross-presented antigen (in the absence of direct infection) is sufficient to prime a broad array of MCMV-specific T cell responses [33]. Likewise, a recent study has shown that vaccination with a spread defective MCMV can prime both T cell and neutralizing antibody responses, and protect susceptible mice against subsequent MCMV challenge [53]. The current study shows, remarkably, that spread defective MCMV continues to elicit effector/effector memory CD8+ T cells over long periods of time. That it may be possible to retain the unique immunogenic properties of CMV in a vaccine vector which is completely unable to cause disease is perhaps the most significant implication of our results. However, memory inflation was only observed when ΔgL was administered systemically. For a prophylactic vaccine it will be necessary to find a route of peripheral administration that would enable the spread defective vaccine to gain access to the cells it needs for latent/persistent infection. For a therapeutic vaccine in cancer patients, a systemic route may be feasible.

## Materials and Methods

### Ethics statement

All animal work was performed in accordance with NIH guidelines and the Animal Welfare Act. The OHSU Department of Comparative Medicine and Division of Animal Resources have full accreditation from the Association for Assessment and Accreditation of Laboratory Animal Care (AAALAC). The experiments were approved by the Institutional Biosafety Committee and the Institutional Animal Care and Use Committee at OHSU.

### Viruses

To produce the MCMV-TK virus, the Thymidine Kinase gene from HSV-1 was inserted into the m157 locus of MCMV using λRED mediated recombination [54]–[56]. Briefly, *TK* from HSV-1 was amplified from a plasmid (kindly provided by David Johnson) and sub-cloned into a second plasmid containing kanamycin flanked by *FRT* recombination sites (kindly provided by Jay Nelson). PCR was performed to generate the *TK-Kan* construct flanked with MCMV sequences in the *m157* region and this PCR product was recombined with wild-type MCMV cloned into a bacterial artificial chromosome (BAC, strain MW97.01 [57]). The final product replaced the entire *m157* coding region with HSV-1 *TK*. Kanamycin was removed by Flp-mediated recombination of the *FRT* sites and the final product was verified by PCR and sequencing. The ΔgL virus has been recently described elsewhere ([33] and Allan et. al. manuscript submitted). Briefly, a 4.2 k.b. construct bearing β-galactosidase was inserted into the middle of the gL coding region from the K181 strain of MCMV, functionally deleting MCMV-gL. Stocks of the ΔgL virus were produced on gL-3T3 cells, which provide gL in *trans* ([33] and Allan et. al. manuscript submitted). Wild-type K181 MCMV and the MW97.01 strain (hereafter called WT-BAC) were used as control, wild-type viruses.

### Mice and infections

BALB/c, BALB/c-SCID mice (CBySmn.CB17-Prkdcscid/J) and C57BL/6 (B6) mice were purchased from Jackson Laboratories. In some cases B6.SJL-Ptprc^a^ Pepc^b^/BoyJ (CD45.1 congenic), originally purchased from the Jackson Labs, were used in place of B6 mice. All studies were approved by the Institutional Biosafety Committee and the Institutional Animal Care and Use Committee at OHSU. For direct infections, unless otherwise indicated mice were injected with 1×10^5^ pfu of MCMV (multiple strains) via the intraperitoneal (i.p.) route or 8×10^4^ pfu into the hind foot pad. In some cases famcyclovir (TEVA Pharmaceuticals USA, Sellersville, PA) was mixed in the drinking water at a concentration of 2 mg/ml and maintained for the indicated amount of time. For indirect infections, infected murine embryonic fibroblasts (MEFs) were injected into B6 mice as described elsewhere [33]. Briefly, 1×10^5^ B6 MEFs were plated overnight in 6 well plates before infection with 3×10^5^ pfu of ΔgL or K181 viruses. After 3 hours, cells were washed twice with PBS, incubated for 60 seconds with 1 ml of citrate buffer (50 mM sodium citrate, 4 mM potassium chloride, pH = 3) and washed twice more with complete media (DMEM+10% FetalPlex−Gemini Bioproducts). Cells were then harvested with trypsin-EDTA (Gibco), washed and resuspended in PBS for injection. B6 mice received approximately 1×10^5^ infected MEFs by i.p. injection. In these experiments, a fraction of the MEFs was checked for infectious virus by plaque assay on gL-3T3s. No plaques were found in any experiment. For infections of BALB/c-SCID mice with wild-type K181 or ΔgL virus, animals were separated into groups based on the amount of injected virus. When any mouse in a group appeared moribund (hunched posture, ruffled fur, swollen eyes and/or sluggishness), all mice in the group were sacrificed. No morbidity was observed in ΔgL-infected mice prior to sacrifice at day 42. Organs were harvested and DNA was prepared for qPCR as described below.

### Plaque assay

To determine the sensitivity of MCMV-TK to acyclovir, 200 plaque forming units of wild-type or MCMV-TK was used to infect BALB-3T3s without centrifugal enhancement in the presence of the indicated amount of acyclovir (Bedford Laboratories). Infected wells were overlaid with 0.375% (w/v) carboxy-methylcellulose and plaques were enumerated 6 days later. To determine viral titers in organs, infected tissue was disrupted by dounce homogenization using a drill. Salivary gland tissue was further disrupted by sonication and debris was removed from all tissue by centrifugation at 2500× g before samples were aliquoted and frozen. Viral titers were determined by plaque assay without centrifugal enhancement using BALB-3T3s (wild-type viruses or MCMV-TK) or gL-3T3s (ΔgL virus).

### Quantitative PCR

To determine amount of viral genome present in infected tissue, organs were disrupted as above and frozen without further processing. DNA was isolated from spleen and salivary gland tissue using the QiaAmp kit (Qiagen) and following the manufacturer's recommended protocol. Quantitative PCR for the MCMV E1 gene was performed using the Taqman Universal PCR Mastermix (Applied Biosystems). The primers and probe have been described elsewhere [33]. To calculate the genome copy number, a standard curve of a plasmid containing the E1 gene from MCMV (pGEM-T-E1) was included in every assay.

### Analysis of antigen specific CD8+ T cells

Antigen specific CD8+ T cells were identified by IFN-γ expression after peptide stimulation or by tetramer staining as described elsewhere [13], [58]. The peptides that were loaded into tetramers or used to stimulate IFN-γ were as follows: M45 - HGIRNASFI; M57 - SCLEFWQRV; m139 - TVYGFCLL; M38 - SSPPMFRV; IE3 - RALEYKNL; IE1 - YPHFMPTNL; m164 - AGPPRYSRI. Tetramers were synthesized by the NIH tetramer core facility (http://www.niaid.nih.gov/reposit/tetramer/overview.html). Samples were acquired on an LSR II flow cytometer (BD Biosciences) and data were analyzed with FlowJo software (Tree Star).

## References

[1] Mendelson M, Monard S, Sissons P, Sinclair J (1996). Detection of endogenous human cytomegalovirus in CD34+ bone marrow progenitors.. J Gen Virol.

[2] Seckert CK, Renzaho A, Tervo HM, Krause C, Deegen P (2009). Liver sinusoidal endothelial cells are a site of murine cytomegalovirus latency and reactivation.. J Virol.

[3] Reddehase MJ, Simon CO, Seckert CK, Lemmermann N, Grzimek NK (2008). Murine model of cytomegalovirus latency and reactivation.. Curr Top Microbiol Immunol.

[4] Sinclair J, Sissons P (2006). Latency and reactivation of human cytomegalovirus.. J Gen Virol.

[5] Kurz SK, Rapp M, Steffens HP, Grzimek NK, Schmalz S (1999). Focal transcriptional activity of murine cytomegalovirus during latency in the lungs.. J Virol.

[6] Simon CO, Holtappels R, Tervo HM, Bohm V, Daubner T (2006). CD8 T cells control cytomegalovirus latency by epitope-specific sensing of transcriptional reactivation.. J Virol.

[7] Karrer U, Sierro S, Wagner M, Oxenius A, Hengel H (2003). Memory inflation: continuous accumulation of antiviral CD8+ T cells over time.. J Immunol.

[8] Karrer U, Wagner M, Sierro S, Oxenius A, Hengel H (2004). Expansion of protective CD8+ T-cell responses driven by recombinant cytomegaloviruses.. J Virol.

[9] Komatsu H, Inui A, Sogo T, Fujisawa T, Nagasaka H (2006). Large scale analysis of pediatric antiviral CD8+ T cell populations reveals sustained, functional and mature responses.. Immun Ageing.

[10] Munks MW, Cho KS, Pinto AK, Sierro S, Klenerman P (2006). Four distinct patterns of memory CD8 T cell responses to chronic murine cytomegalovirus infection.. J Immunol.

[11] Northfield J, Lucas M, Jones H, Young NT, Klenerman P (2005). Does memory improve with age? CD85j (ILT-2/LIR-1) expression on CD8 T cells correlates with ‘memory inflation’ in human cytomegalovirus infection.. Immunol Cell Biol.

[12] Sierro S, Rothkopf R, Klenerman P (2005). Evolution of diverse antiviral CD8+ T cell populations after murine cytomegalovirus infection.. Eur J Immunol.

[13] Snyder CM, Cho KS, Morrison EL, van Dommelen S, Shellam GR (2008). Memory Inflation During Chronic Viral Infection is Maintained by Continuous Production of Short-Lived Functional T Cells.. Immunity.

[14] Sylwester AW, Mitchell BL, Edgar JB, Taormina C, Pelte C (2005). Broadly targeted human cytomegalovirus-specific CD4+ and CD8+ T cells dominate the memory compartments of exposed subjects.. J Exp Med.

[15] Hadrup SR, Strindhall J, Kollgaard T, Seremet T, Johansson B (2006). Longitudinal studies of clonally expanded CD8 T cells reveal a repertoire shrinkage predicting mortality and an increased number of dysfunctional cytomegalovirus-specific T cells in the very elderly.. J Immunol.

[16] Koch S, Larbi A, Ozcelik D, Solana R, Gouttefangeas C (2007). Cytomegalovirus infection: a driving force in human T cell immunosenescence.. Ann N Y Acad Sci.

[17] Ouyang Q, Wagner WM, Wikby A, Walter S, Aubert G (2003). Large numbers of dysfunctional CD8+ T lymphocytes bearing receptors for a single dominant CMV epitope in the very old.. J Clin Immunol.

[18] Ouyang Q, Wagner WM, Zheng W, Wikby A, Remarque EJ (2004). Dysfunctional CMV-specific CD8(+) T cells accumulate in the elderly.. Exp Gerontol.

[19] Ouyang Q, Wagner WM, Voehringer D, Wikby A, Klatt T (2003). Age-associated accumulation of CMV-specific CD8+ T cells expressing the inhibitory killer cell lectin-like receptor G1 (KLRG1).. Exp Gerontol.

[20] Vescovini R, Biasini C, Fagnoni FF, Telera AR, Zanlari L (2007). Massive load of functional effector CD4+ and CD8+ T cells against cytomegalovirus in very old subjects.. J Immunol.

[21] Appay V, Dunbar PR, Callan M, Klenerman P, Gillespie GM (2002). Memory CD8+ T cells vary in differentiation phenotype in different persistent virus infections.. Nat Med.

[22] Thimme R, Appay V, Koschella M, Panther E, Roth E (2005). Increased expression of the NK cell receptor KLRG1 by virus-specific CD8 T cells during persistent antigen stimulation.. J Virol.

[23] Gamadia LE, van Leeuwen EM, Remmerswaal EB, Yong SL, Surachno S (2004). The size and phenotype of virus-specific T cell populations is determined by repetitive antigenic stimulation and environmental cytokines.. J Immunol.

[24] van Leeuwen EM, de Bree GJ, Remmerswaal EB, Yong SL, Tesselaar K (2005). IL-7 receptor alpha chain expression distinguishes functional subsets of virus-specific human CD8+ T cells.. Blood.

[25] Lang A, Brien JD, Nikolich-Zugich J (2009). Inflation and long-term maintenance of CD8 T cells responding to a latent herpesvirus depend upon establishment of latency and presence of viral antigens.. J Immunol.

[26] Elion GB, Furman PA, Fyfe JA, de Miranda P, Beauchamp L (1977). Selectivity of action of an antiherpetic agent, 9-(2-hydroxyethoxymethyl) guanine.. Proc Natl Acad Sci U S A.

[27] Eizuru Y, Minamishima Y, Hirano A, Kurimura T (1978). Replication of mouse cytomegalovirus in thymidine kinase-deficient mouse cells.. Microbiol Immunol.

[28] Muller MT, Hudson JB (1977). Thymidine kinase activity in mouse 3T3 cells infected by murine cytomegalovirus (MCV).. Virology.

[29] Arase H, Mocarski ES, Campbell AE, Hill AB, Lanier LL (2002). Direct recognition of cytomegalovirus by activating and inhibitory NK cell receptors.. Science.

[30] Smith HR, Heusel JW, Mehta IK, Kim S, Dorner BG (2002). Recognition of a virus-encoded ligand by a natural killer cell activation receptor.. Proc Natl Acad Sci U S A.

[31] Lang A, Brien JD, Messaoudi I, Nikolich-Zugich J (2008). Age-related dysregulation of CD8+ T cell memory specific for a persistent virus is independent of viral replication.. J Immunol.

[32] LeBlanc RA, Pesnicak L, Godleski M, Straus SE (1999). The comparative effects of famciclovir and valacyclovir on herpes simplex virus type 1 infection, latency, and reactivation in mice.. J Infect Dis.

[33] Snyder CM, Allan JE, Bonnett EL, Doom CM, Hill AB (2010). Cross-Presentation of a Spread-Defective MCMV Is Sufficient to Prime the Majority of Virus-Specific CD8+ T Cells.. PLoS One.

[34] Heldwein EE, Krummenacher C (2008). Entry of herpesviruses into mammalian cells.. Cell Mol Life Sci.

[35] Jabbari A, Harty JT (2006). Secondary memory CD8+ T cells are more protective but slower to acquire a central-memory phenotype.. J Exp Med.

[36] Masopust D, Ha SJ, Vezys V, Ahmed R (2006). Stimulation history dictates memory CD8 T cell phenotype: implications for prime-boost vaccination.. J Immunol.

[37] Hsu KM, Pratt JR, Akers WJ, Achilefu SI, Yokoyama WM (2009). Murine cytomegalovirus displays selective infection of cells within hours after systemic administration.. J Gen Virol.

[38] Balthesen M, Dreher L, Lucin P, Reddehase MJ (1994). The establishment of cytomegalovirus latency in organs is not linked to local virus production during primary infection.. J Gen Virol.

[39] Kurz SK, Reddehase MJ (1999). Patchwork pattern of transcriptional reactivation in the lungs indicates sequential checkpoints in the transition from murine cytomegalovirus latency to recurrence.. J Virol.

[40] Katz JP, Bodin ET, Coen DM (1990). Quantitative polymerase chain reaction analysis of herpes simplex virus DNA in ganglia of mice infected with replication-incompetent mutants.. J Virol.

[41] Kayhan B, Yager EJ, Lanzer K, Cookenham T, Jia Q (2007). A replication-deficient murine gamma-herpesvirus blocked in late viral gene expression can establish latency and elicit protective cellular immunity.. J Immunol.

[42] Moser JM, Farrell ML, Krug LT, Upton JW, Speck SH (2006). A gammaherpesvirus 68 gene 50 null mutant establishes long-term latency in the lung but fails to vaccinate against a wild-type virus challenge.. J Virol.

[43] Tibbetts SA, Suarez F, Steed AL, Simmons JA, Virgin HWt (2006). A gamma-herpesvirus deficient in replication establishes chronic infection in vivo and is impervious to restriction by adaptive immune cells.. Virology.

[44] Bevan IS, Sammons CC, Sweet C (1996). Investigation of murine cytomegalovirus latency and reactivation in mice using viral mutants and the polymerase chain reaction.. J Med Virol.

[45] Mercer JA, Wiley CA, Spector DH (1988). Pathogenesis of murine cytomegalovirus infection: identification of infected cells in the spleen during acute and latent infections.. J Virol.

[46] Goodrum FD, Jordan CT, High K, Shenk T (2002). Human cytomegalovirus gene expression during infection of primary hematopoietic progenitor cells: a model for latency.. Proc Natl Acad Sci U S A.

[47] Reeves MB, MacAry PA, Lehner PJ, Sissons JG, Sinclair JH (2005). Latency, chromatin remodeling, and reactivation of human cytomegalovirus in the dendritic cells of healthy carriers.. Proc Natl Acad Sci U S A.

[48] Dutko FJ, Oldstone MB (1981). Cytomegalovirus causes a latent infection in undifferentiated cells and is activated by induction of cell differentiation.. J Exp Med.

[49] Liu T, Khanna KM, Chen X, Fink DJ, Hendricks RL (2000). CD8(+) T cells can block herpes simplex virus type 1 (HSV-1) reactivation from latency in sensory neurons.. J Exp Med.

[50] Munks MW, Pinto AK, Doom CM, Hill AB (2007). Viral interference with antigen presentation does not alter acute or chronic CD8 T cell immunodominance in murine cytomegalovirus infection.. J Immunol.

[51] Hansen SG, Ford JC, Lewis MS, Ventura AB, Hughes CM (2011). Profound early control of highly pathogenic SIV by an effector memory T-cell vaccine.. Nature.

[52] Hansen SG, Vieville C, Whizin N, Coyne-Johnson L, Siess DC (2009). Effector memory T cell responses are associated with protection of rhesus monkeys from mucosal simian immunodeficiency virus challenge.. Nat Med.

[53] Mohr CA, Arapovic J, Muhlbach H, Panzer M, Weyn A (2010). A spread deficient cytomegalovirus for assessment of first target cells in vaccination.. J Virol.

[54] Yu D, Ellis HM, Lee EC, Jenkins NA, Copeland NG (2000). An efficient recombination system for chromosome engineering in Escherichia coli.. Proc Natl Acad Sci U S A.

[55] Messerle M, Crnkovic I, Hammerschmidt W, Ziegler H, Koszinowski UH (1997). Cloning and mutagenesis of a herpesvirus genome as an infectious bacterial artificial chromosome.. Proc Natl Acad Sci U S A.

[56] Lee EC, Yu D, Martinez de Velasco J, Tessarollo L, Swing DA (2001). A highly efficient Escherichia coli-based chromosome engineering system adapted for recombinogenic targeting and subcloning of BAC DNA.. Genomics.

[57] Wagner M, Jonjic S, Koszinowski UH, Messerle M (1999). Systematic excision of vector sequences from the BAC-cloned herpesvirus genome during virus reconstitution.. J Virol.

[58] Snyder CM, Loewendorf A, Bonnett EL, Croft M, Benedict CA (2009). CD4+ T cell help has an epitope-dependent impact on CD8+ T cell memory inflation during murine cytomegalovirus infection.. J Immunol.

